# Supplementary material for: Feasibility and Early Outcomes of a Parent Training Intervention to Engage Parents in Children’s Media Education
Source: Healthcare (Basel). 2023 Jul 26;11(15):2130. doi: 10.3390/healthcare11152130 (PMC10418340; doi:10.3390/healthcare11152130)
Supplement: Supplementary file 1 [file healthcare-11-02130-s001.zip › healthcare-2383851-supplementary.pdf]

Supplementary material

To support the qualitative data, we administered some quantitative questionnaires. In particular, we wanted to measure the levels of problematic and openness of parent-child communication, the frequency of mediation strategies, and the risks experienced by the children. Since ACT-based exercises are also aimed at reducing parental stress, we chose to measure this construct as well.

Parenting Stress Index - Short Form (PSI-SF), that derives directly from *the Parenting Stress Index* [1]. The Italian validation of the PSI-SF was conducted by Guarino and colleagues [2]. The measure assesses three dimensions: (1) Parental Distress, (2) Parent-Child Dysfunctional Interaction, and (3) Difficult Child. Examples of items are, "My son usually wakes up in a bad mood," "I feel that my son is easily irritable or in a bad mood," "I feel trapped in my responsibilities as a parent," "Since I had my son, I can't do new and different things."

Parent Adolescents Communication Scale [3], that assesses the levels of problematic or open communication between parents and children. It consists of 20 items measuring the quality of communication between adolescent and parent. Each item describes behavior, situations, or facts linked to the quality of parent-adolescent communication. Participants express their degree of agreement with what is stated on each item using a five-option Likert scale. Examples of item are: “I can discuss my beliefs with my child without feeling restrained or embarrassed”, "I openly show affection to my child”, “There are topics I avoid discussing with my child”.

Checklists derived from the **Net Children Go Mobile parent form** [4] regarding mediation strategies and risks experienced online.

All questionnaires were completed after creating a self-produced anonymous code that would allow us to link pre-intervention responses to post-intervention responses.

Results

Due to the low rate of participants answering the quantitative measures, the quantitative data only provided information about trends and did not reach the significance at the statistical tests. Results about Communication showed a slight trend of increase in Openness and decrease in the Problematicity of communication (Table S1).

Table S1. *T*-test results the quality of parents-children communication: differences between pre and post administration.

	M(*SD*)	*t(df)*	*Sig.*
Communication Openness PRE	38.2(6.4)	-0.72(6)	0.49
Communication Openness POST	40.2(3.8)		
Communication problems PRE	24(7.5)	-0.10(6)	0.92
Communication problems POST	23.2(8.6)		

With respect to different mediation strategies, a slight increase in the frequency of active mediation strategies (*I sit with my child when he/she is on the Internet, I talk with him/her about what may be happening online, I share my concerns with him/her, We create shared rules* etc.) and a slight decrease in children's online risks perceived by parents emerged (Table S2).

Table S2. Mediation strategies and online risks t-test results: differences between pre and post somministration.

	M(*SD*)	*t(df)*	*Sig.*
Active mediation strategies PRE	3.2(2.5)	-1.2(6)	0.24
Active mediation strategies POST	5.8(1.3)		
Restrictive mediation strategies PRE	7(3.8)	-0.47(6)	0.65
Restrictive mediation strategies POST	7(2.9)		
Children online risks PRE	16.2(1.4)	0.00(6)	1
Children online risks POST	15.6(2.0)		

Regarding the parent stress dimensions, a slight reduction in the average of two of the three dimensions ("Parental Distress" and "Difficult Child"), and a tendency to increase in the average of the dimension "Dysfunctional Interaction" emerged (Table S3).

Table S3. PSI questionnaire t-test results: differences between pre and post somministration.

	M(*SD*)	*t(df)*	*Sign.*
Parental Distress PRE	31(16.5)	0.290(6)	0.78
Parental Distress POST	28.8(16.2)		
Dysfunctional Interaction PRE	25.2(12.2)	-1.5(6)	0.18
Dysfunctional Interaction POST	27(15.7)		
Difficult Child PRE	24(14.8)	-0.64(6)	0.54
Difficult Child POST	22.8(18.1)		

References

[1]Abidin, R. R. (1995). The Parenting Stress Index (3rd ed.). Professional manual. Odessa, FL: Psychological Assessment Resources, Inc.

[2] Guarino, A., Di Blasio, P., D’Alessio, M., Camisasca, E., & Serantoni, G. (2008). Parenting stress index—Forma breve. *Firenze: Giunti Organizzazioni Speciali*.

[3] Barnes, H. L., & Olson, D. H. (1985). Parent-Adolescent communication and the Circumplex Model. *Child Development*, 56(2), 438–447. https://doi.org/10.2307/1129732

[4] Mascheroni, G., & Ólafsson, K. (2014). Net children go mobile: Risks and opportunities. http://eprints.lse.ac.uk/55798/1
